# Barriers and Facilitators to Implementing an Evidence-Based Community Health Worker Model

**DOI:** 10.1001/jamahealthforum.2024.0034

**Published:** 2024-03-08

**Authors:** Simone H. Schriger, Molly Knowles, Talia Daglieri, Shreya Kangovi, Rinad S. Beidas

**Affiliations:** 1 Department of Psychology, University of Pennsylvania, Philadelphia; 2 Perelman School of Medicine, University of Pennsylvania, Philadelphia; 3 Penn Center for Community Health Workers, Philadelphia, Pennsylvania; 4 IMPaCT Care, Philadelphia, Pennsylvania; 5 Department of Medical Social Sciences, Feinberg School of Medicine, Northwestern University, Chicago, Illinois

## Abstract

**Importance:**

Community health worker (CHW) programs may improve health outcomes, increase quality of life, and reduce hospitalizations and cost of care. However, knowledge is limited on the barriers and facilitators associated with scaling evidence-based CHW programs to maximize their public health outcomes.

**Objective:**

To identify barriers and facilitators to implementing an evidence-based CHW model.

**Design, Setting, and Participants:**

This qualitative study examined perspectives of Individualized Management of Person-Centered Targets (IMPaCT) program staff (health system leaders, program managers, and community health workers) and patients receiving the intervention between March 9, 2020, and July 22, 2021, at 5 institutionally and geographically diverse health systems across the US. The collected data were analyzed between December 1, 2021, and April 27, 2022. Program staff were recruited via purposive sampling, and patients were recruited via convenience sampling.

**Intervention:**

The disease-agnostic IMPaCT CHW model includes a standardized implementation approach and a structured set of theory-informed intervention components to create and achieve individualized action plans.

**Main Outcomes and Measures:**

Interview guides were informed by the Consolidated Framework for Implementation Research. A rapid qualitative analytic technique was used to identify key themes, which were categorized into barriers and facilitators associated with framework ecological domains.

**Results:**

Of a total 41 individuals invited, 39 agreed to participate (95% response rate; mean [SD] age, 45.0 [12.6] years; 30 women). General barriers included economic and policy constraints, including insufficient funding for CHW programs, clinical integration challenges, and CHW difficulty with maintaining boundaries. Program-specific barriers included insufficiently tailored materials for certain populations and upfront and ongoing program costs. General facilitators included CHWs’ interpersonal skills and life experiences. Program-specific facilitators included the model’s strong evidence base, supportive implementation team, and program design that enabled relationship building and engagement. Additional themes were cited as both barriers and facilitators, including the COVID-19 pandemic, organizational leadership, IMPaCT training, and program fidelity.

**Conclusions and Relevance:**

These findings suggest growing recognition of the importance of CHWs to improving health equity and population health. Barriers identified point to important policy and practice implications for CHW programs more broadly, including the need for continued attention to improving clinical integration and the need for sustainable program financing to preserve the longevity of this workforce.

## Key Points

**Question:**

What barriers and facilitators are associated with implementing an evidence-based community health worker (CHW) model?

**Findings:**

In this qualitative study examining the perspectives of 39 individuals involved in implementing a CHW model, salient implementation barriers included difficulties with clinical integration and financial challenges, and facilitators included the model’s emphasis on relationships and its evidence-based components and strategies.

**Meaning:**

These findings suggest that implementation barriers and facilitators may offer important policy and practice implications for how best to support CHW programs.

## Teaser

This qualitative study identifies barriers and facilitators to implementing an evidence-based community health worker model.

## Introduction

Community health workers (CHWs) are frontline public health workers who are trusted members of the communities they serve.^1^ Broadly, CHW programs have been shown to improve a wide range of outcomes, including population health,^2,3,4,5^ patient experience of care,^2,6,7^ health equity,^8,9,10^ and reduced health care costs.^11,12,13,14,15,16^ The Individualized Management of Person-Centered Targets (IMPaCT) model, tested in 3 randomized trials,^2,6,7^ has been found to improve health outcomes, increase quality of care, and reduce total hospital days, while demonstrating a $2.47:1 return on investment within 1 year. Given the effectiveness of this model, elucidating potential barriers and facilitators to implementation is an important next step in the process of scaling this approach in health care delivery and can also produce generalizable insights for other evidence-based CHW programs.

The IMPaCT model uses a standardized implementation approach spanning 3 stages: preimplementation (partner-engaged planning, feasibility assessment, and adaptation), implementation (recruitment and hiring support, comprehensive training, standardized work practice manuals, and ongoing technical assistance to address implementation barriers), and sustainment (ongoing learning opportunities for CHWs and program leadership and tailored expansion support). Additionally, IMPaCT includes a structured set of theory-informed intervention components: community-based CHW hiring strategies (eg, recruitment through advertisements and presentations provided at local community centers, churches, and retail stores); standardized intervention durations, caseloads, and protocols for supervision and safety; clinical integration strategies that balance time spent in clinical and community settings (eg, physician’s office, patient’s home); ongoing performance assessment; and adaptability across diseases, settings, and populations. Unlike many CHW models, IMPaCT is disease-agnostic. When delivering IMPaCT, CHWs prioritize building strong relationships using person-centered practices and use a semistructured interview guide to understand patients’ priorities and the factors associated with their health, collaborate with patients to create individualized goals and action plans, and provide tailored support to patients in executing those action plans.

While IMPaCT is a leading evidence-based CHW model nationally,^2,6,7,11^ factors associated with its implementation have yet to be studied. To achieve maximal public health benefit and to contribute to the broader literature on implementing, scaling, and sustaining evidence-based CHW programs, examination of implementation barriers and facilitators (ie, determinants) is needed. The Consolidated Framework for Implementation Research (CFIR)^17,18^ provides a theoretical framework to guide understanding of implementation determinants across multiple domains. The primary aim of this study was to understand implementation determinants by soliciting perspectives of individuals from multiple constituent groups involved in IMPaCT implementation at 5 institutionally and geographically diverse health care settings.

## Methods

All procedures used for this qualitative study were approved by the institutional review board at the University of Pennsylvania. All participants provided verbal consent for participation and the sharing of their deidentified data for reasearch purposes, and no participants withdrew from the study following informed consent. The Standards for Reporting Qualitative Research (SRQR) were followed for reporting results.^19^

### Participants

Participants included multilevel program staff (health system leaders, program managers, and CHWs) and patients at 5 health systems that had launched the IMPaCT program in the previous year. Sites were selected based on geographic and institutional diversity (Table 1). Our target sample size was informed by empirical literature on the sample size needed to reach thematic saturation. Previous literature suggested that saturation is typically reached within 9 to 17 interviews.^20^ We anticipated that a sample size of 12 interviewees per constituent group would be a reasonable target. We used purposive sampling to recruit program staff and convenience sampling to recruit patients. Health system leaders were identified by the IMPaCT implementation team (M.K., T.D., and S.K.) and connected to the implementation evaluation team (S.H.S. and R.S.B.) via email. All health system leaders were asked to participate. Leaders then connected the implementation evaluation team to program managers via email. All managers were invited to participate. Managers were asked to identify 1 champion CHW (described as particularly enthusiastic, diligent, committed to their role, and motivated to go above and beyond). Up to 3 additional CHWs at each site who had been in their roles for at least 6 months were also invited at random to participate. Finally, CHWs provided a list of patients who consented to participate in an interview. Patients were randomly selected from this list and invited to participate via phone, with up to 3 participating from each site. While we anticipated that approximately 2 to 4 CHWs and patients per site would be sufficient to reach thematic saturation, we remained open to conducting additional interviews within these groups if saturation was not reached.

**Table 1.** Site Characteristics

Site^a^	US region	Institution type	No. of participants			
			Leaders (n = 11)	Managers (n = 4)	CHWs (n = 12)	Patients (n = 12)
A	Northeast	Urban public hospital	3	1	2	2
B	South	Integrated health system	2	1	1	2
C	Northeast	Integrated health system	2	1	2	2
D	West	Federally qualified health center	2	1	4	3
E	West	Integrated payer-provider	2	0^b^	3	3

Abbreviation: CHW, community health worker.

^a^ Sites labeled here do not correspond to the sites referenced in Table 3 for participant anonymity due to small samples size.

^b^ At the time of interviewing, the previous manager had left, and the role had not yet been filled.

### Procedure

The evaluation team developed a semistructured interview guide for each participant group based on the 5 ecological domains of the original CFIR^17^ (interview guides provided in eMethods 1 in Supplement 1). Interviews with program staff emphasized each stage of IMPaCT implementation, while interviews with patients focused on their experiences as intervention recipients.

Although we used the original CFIR^17^ to develop interview guides, an updated CFIR^18^ was published prior to data analysis. We consulted both versions and use language from the updated CFIR^18^ in this article. The 5 CFIR domains include innovation (eg, the IMPaCT intervention), outer setting (eg, broader sociopolitical context), inner setting (eg, specific organization in which IMPaCT is being implemented), individuals (eg, program staff implementing IMPaCT, patients participating in the program), and implementation process (eg, strategies used to implement IMPaCT).

Three implementation evaluation team members (not involved in the development of IMPaCT and including S.H.S. and R.S.B.) conducted interviews between March 9, 2020, and July 22, 2021. Interviewers had a variety of training backgrounds (advanced clinical psychology doctoral student; implementation scientist and psychologist; advanced baccalaureate student research assistant) and had no previous relationship with interviewees. Interviews were conducted via telephone and audio recorded and lasted approximately 30 to 60 minutes. Interviewees were briefed on confidentiality at the start of each interview and informed that the interviewer was not part of the IMPaCT implementation team. At the end of each interview, participants were asked to provide demographic information, including their age, sex, race, and ethnicity. Participants self-identified their race and ethnicity based on standard National Institutes of Health categories (American Indian or Alaska Native, Asian, Black or African American, Hispanic, Native Hawaiian or Pacific Islander, White) and could select multiple options or decline to answer. Race and ethnicity were included among other demographics to better capture the representativeness of the sample. Interviewees received $30 for participating. Interviews were transcribed for analysis, and interviewers met regularly to discuss themes and thematic saturation.

### Analysis

The collected data were analyzed between December 1, 2021, and April 27, 2022. A rapid qualitative analytic technique^21,22,23,24^ was used to identify key themes within and across interviewee groups. A structured summary template was created using key elements from the interview guides to organize and condense data based on CFIR domains. We used an integrated (ie, both deductive and inductive) approach to data analysis. While the 5 ecological domains of the CFIR were used to organize interview guides and structure themes, we used an inductive, emergent, open-coding approach to the themes themselves without any predetermined categorization. Three implementation evaluation team members (including S.H.S.) participated in the initial coding of interviews, during which transcripts were synthesized into summary sheets. Team members kept memos during the analytic process and met weekly to discuss insights and patterns and to prevent drift. In line with best practices, at the start of the analytic process, all coders double-coded the same subset of transcripts (4 interviews [10%]) to establish a consistent coding practice and review any discrepancies that arose as well as address questions about the coding process. Coders then evenly divided and coded the remaining interviews. Once all interviews were coded, completed summary sheets were transferred into matrix displays by the main coder (S.H.S.) and categorized into barriers and facilitators within each CFIR domain for each respondent group. Matrices for each group were then compared to determine cross-cutting themes and explore differences across groups. Once all interviews were coded, completed summary sheets were transferred into matrix displays within Microsoft Excel, version 16.81 (Microsoft Corporation) by the main coder (S.H.S.) and categorized.

## Results

Of the 41 individuals contacted, 39 (11 leaders, 4 managers, 12 CHWs, 12 patients) agreed to participate (95% response rate), including 30 women and 9 men. The mean (SD) age of the sample was 45.0 (12.6) years. In total, 15 participants identified as Hispanic and/or Latino, and 1 identified as American Indian or Alaska Native, 2 as Asian, 10 as Black or African American, and 27 as White (some participants selected >1 race) (Table 2). We found sufficient overlap among responses to suggest that thematic saturation had been reached and, thus, did not conduct additional interviews. Here, we present the most salient themes from each CFIR domain. Salience was determined by extent of endorsement both within groups (eg, noted by multiple CHWs) and across groups (eg, noted by both CHWs and managers). We indicate whether each theme was viewed by participants as a barrier, facilitator, or, in some cases, both. We present aggregated results across participant groups because, apart from perspectives on clinical integration, there were no divergent findings between groups. Themes and illustrative quotes are provided in Table 3, a detailed description of themes is provided in eMethods 2 in Supplement 1, and additional quotes are provided in eMethods 3 in Supplement 1. Table 4 details factors across the domains of the CFIR that may lead to CHW burnout.

**Table 2.** Participant Demographic Characteristics

Variable	No. of participants			
	Leaders (n = 11)	Managers (n = 4)	CHWs (n = 12)	Patients (n = 12)
Age, mean (SD), y	40.5 (7.8)	35.0 (6.4)	39.7 (9.4)	57.9 (12.2)
Sex				
Female	8	4	10	8
Male	3	0	2	4
Ethnicity				
Hispanic and/or Latino	3	3	6	3
Non-Hispanic and/or non-Latino	8	1	6	9
Race^a^				
American Indian or Alaska Native	0	0	0	1
Asian	1	0	0	1
Black or African American	0	1	4	5
Native Hawaiian or Pacific Islander	0	0	0	0
White	10	3	8	6

Abbreviation: CHW, community health worker.

^a^ Because participants could select multiple responses, percentages may not sum to 100.

**Table 3.** Domains, Themes, and Illustrative Quotes

Domain or theme	Explanation	Barrier (−), facilitator (+), or both (−/+)	Illustrative quote
Innovation			
Program cost	High program and associated costs (certification, recommended software) were a concern and potential barrier to sustainment.	−	“It’s pricey and hard to sustain over a long period of time.” (Leader 2, site 5)
Applicability to specific marginalized populations	Program materials, particularly program manuals, lacked applicability to certain populations (eg, undocumented and non–English-speaking patients).	−	“I remember the Spanish versions being difficult to read. Like, somebody tried to translate the English to the Spanish and it didn’t really flow very well.” (CHW 1, site 5)
Evidence base of program	The robust evidence base that supported IMPaCT’s effectiveness facilitated buy-in.	+	“It’s so nice to be able to build a data-driven program vs just like going in the dark with all these interventions.” (Leader 1, site 1)
Program design	The IMPaCT model’s set of structured components and implementation strategies facilitated program success.	+	“I just really love this model, and I’m really grateful that I was trained under this model because I used to work as a CHW back in the day, and I used to oversee CHWs…really didn’t have no model. So this was more of a structure, I’m able to provide my staff the real support they need. I love overall the model, the way it helps [me] help not only my staff but my patients and the community overall.” (Manager, site 5)
Outer setting			
Economic and political climate	Current economic conditions have strained funding and led to greater patient needs, and legislation affecting CHW programs has created additional implementation challenges.	−	“There’s a phrase going around, ‘no money, no mission.’ That’s one of the struggles that we’re having.” (Leader 1, site 5)
COVID-19 pandemic	The pandemic has exacerbated existing challenges (eg, financial strain) and created new ones (eg, shift to telehealth), although in some cases, it has facilitated implementation (eg, led to creative types of interventions and delivery modalities).	−/+	“It’s harder to kinda connect with patients over the phone. We’re so used to going to home visits, being at the doctor, being at the bedside when they’re getting discharged, so it’s a little bit harder to connect because I’m just a phone person now. They don’t see who I am.” (CHW 1, site 1)
			“It definitely made a difference, because I feel like now we’re closer. During the pandemic we got a little closer where before, we were kind of like stick to our schedule. So I think that has been a benefit.” (Manager, site 3)
Inner setting			
Clinical integration	Despite IMPaCT’s emphasis on clinical integration strategies, many CHWs faced significant challenges with integration and at times felt misunderstood and disrespected by other allied health professionals.	−	“We’re looked at as not important in the role that we play.” (CHW 1, site 5)
Individuals			
Leadership at organization	Executive buy-in was fundamental to implementation success, while unsupportive leaders challenged program longevity.	−/+	“Today it feels like they [leadership at organization] still don’t know much about it....Even though there was this big meeting and those periodic emails…I feel like the understanding of the model today isn’t that strong.” (Leader 2, site 5)
			“I think it was very helpful to have someone who knew the importance of having a CHW and that definitely took the risk of saying we should bring CHWs into our health care system.” (Manager, site 3)
Characteristics of CHWs	Personal characteristics of CHWs were highlighted as the heart of IMPaCT and a potent implementation facilitator.	+	“Because she was Latina, she was able to understand my diet that I eat. Whereas to an American, they really don’t understand that we gotta have our tortillas, we gotta have our beans, we gotta have our rice. And she was able to have me work around my normal diet to where I could eat something else instead.” (Patient 3, site 5)
IMPaCT implementation team	The knowledge, experience, and support of the IMPaCT implementation team was fundamental to successful program implementation.	+	“The folks on the IMPaCT team, you get the sense that they are operators…they’re not the sort of staid academic type that is perhaps a bit too rigid about what real-world conditions might look like.” (Leader 3, site 1)
Implementation process			
Program boundaries	CHWs faced difficulties with program boundaries, particularly in the context of the pandemic. Patients often contacted CHWs beyond the program end date, and CHWs often felt pulled to continue providing support.	−	“They don’t want to talk to anybody else; they just want to talk to you. And so, you become their therapist, you know? And even though we kind of push, ‘You know, we have this program, you can talk to this person,’…they’re just like, ‘No, it’s fine. I’ll just talk to you.’” (CHW 3, site 5)
Training	IMPaCT training was lauded by participants across groups. However, gaps in training, particularly in the realm of mental health, created implementation challenges.	−/+	“We don’t have that training, so we don’t know, other than listening and giving advice, we don’t know what else to do....They said, ‘If you could get another training or 1 more resource, what would it be?’ And we all said, ‘Mental health.’” (CHW 1, site 5)
			“It was amazing…it made me feel so comfortable taking on this role as a CHW. It made me feel so confident, especially all the role plays we did where we can see all the potential situations with the patient....I’ve never seen any better training for any other jobs. It made me want to be a CHW trainer, honestly.” (CHW 2, site 1)
Fidelity	Fidelity to core program components and IMPaCT recommendations was desired by participants. However, funding and procedural constraints created implementation challenges and prevented fidelity to certain model components.	−/+	“Our process looks a lot different than the IMPaCT model’s recommendations just because of our [human resources] restrictions.” (Manager, site 2)
			About IMPaCT-recommended hiring practices: “I really used most of the materials that IMPaCT had developed to plan a meet and greet with people in the community….I found it really useful and different than any of the recruitment processes I had either led or been a part of myself, definitely a unique approach to identifying the qualities and traits of individuals as opposed to, necessarily, their skill sets or past work….[L]ooking back, who we ended up hiring and how they differ a little bit from other [agency name] staff members that are in similar roles across the system, I really attribute that to the different approach we took to recruit them.” (Leader 2, site 1)

Abbreviations: CHW, community health worker; IMPaCT, Individualized Management of Person-Centered Targets.

**Table 4.** Factors Across Domains of the CFIR That May Lead to CHW Burnout

CFIR domain	Description of domain within IMPaCT model	Association with CHW burnout
Innovation	IMPaCT intervention	Materials that require tailoring may fall on CHWs due to their deeper understanding of their patients. The IMPaCT model heavily emphasizes relationships (ie, prioritizes depth and length of connection between patient and CHW).
Outer setting	Broader sociopolitical context in which organizations implementing IMPaCT operate	Budget cuts and unstable funding may result in job precarity. The CHWs may experience the same social determinants of health as their patients and have the same stressors associated with the economic climate and COVID-19 pandemic.
Inner setting	Specific organization in which IMPaCT is being implemented	The CHWs may face frequent challenges with clinical integration, including disrespect from other health care professionals, myths, distrust, confusion about their role, and criticism for lack of credibility.
Individuals	Individuals involved in the implementation of IMPaCT	The CHWs are self-selected helpers with qualities including being passionate, caring, supportive, and highly motivated to care for others, which may be associated with burnout.
Implementation process	Strategies used to implement IMPaCT	Lack of boundaries with patients may result in ever-increasing caseloads and difficulty taking time off; lack of mental health training may result in CHWs feeling ill equipped for certain situations.

Abbreviations: CFIR, Consolidated Framework for Implementation Research; CHW, community health worker; IMPaCT, Individualized Management of Person-Centered Targets.

### Innovation

Participants cited high upfront and ongoing program costs as an implementation barrier. Additionally, although materials were designed to be flexibly adapted, some interviewees felt that they were not sufficiently applicable to specific populations (eg, undocumented patients, non-English speakers). At times, CHWs were called upon to assist with adapting materials given their deep understanding of patient populations. The IMPaCT model’s strong empirical support and its highly structured design were cited as strong implementation facilitators.

### Outer Setting

The broader national economic and political climates were cited as a barrier to implementation both in terms of their association with implementing IMPaCT (eg, budget cuts and limited Medicaid and Medicare reimbursement) and with the patient populations served by IMPaCT (eg, immigration policies). Interviewees also described how the acute period of the COVID-19 pandemic exacerbated existing challenges (eg, increased financial strain and widened disparities) and created new challenges (eg, shift to a virtual format). However, participants also noted that this acute period facilitated greater team camaraderie and created opportunities for new patient service types and modalities.

### Inner Setting

Clinical integration, or the extent to which CHWs were able to embed themselves within clinical settings and collaborate with other health care professionals, was cited as a barrier. Despite IMPaCT’s emphasis on facilitating clinical integration, CHWs and other program staff reported disrespect and skepticism of the CHW role from other health care professionals (eg, social workers and case managers). In contrast, patients did not report perceiving clinical integration challenges, describing their care team as a united front.

### Individuals

Organizational leadership buy-in was viewed as highly related to implementation success; unsupportive leadership was hard to overcome, while supportive leadership strongly facilitated successful program implementation. Interviewees across leader, manager, and patient groups lauded CHWs for their empathy, loyalty, genuineness, trustworthiness, and passion, among other qualities (word cloud of frequently used descriptors provided in the eFigure in Supplement 1). The IMPaCT implementation team members were praised for their enthusiasm, knowledge, and dedication and cited as instrumental to implementation success.

### Implementation Process

Participants highlighted challenges related to maintaining professional boundaries, which were exacerbated by the pandemic. Some CHWs struggled to end relationships with patients, who often continued to contact them beyond their official program end date, and some reported reluctance to take time off from work due to concern for patients who expressed lack of comfort with other health care professionals. Overall, IMPaCT training was highly praised, although several CHWs indicated a need for additional training in mental health. Fidelity to the IMPaCT model was cited as both a barrier and facilitator. Although system constraints prohibited some sites from achieving fidelity to certain model components (particularly, recommended documentation software and hiring practices), participants typically indicated that fidelity to the IMPaCT model facilitated program success.

## Discussion

This qualitative study examined barriers and facilitators to implementing IMPaCT, a leading evidence-based CHW model, across 5 institutionally and geographically diverse health systems. Although interviews were specifically focused on IMPaCT, this model was developed based on best practices within the literature and shares characteristics with many other evidence-based CHW programs.^25,26,27^ Therefore, we view many of these findings as relevant to programs beyond IMPaCT. Our findings add to the limited but growing literature on US-based CHW programs, which operate within a unique set of structural constraints and challenges, including regulatory matters related to credentialing and financing. One key finding was that all constituent groups identified barriers experienced by CHWs across ecological domains, signaling the importance of attending to the needs of this emerging workforce within health care delivery. These results may guide future research and policy development and may aid in long-term sustainment of IMPaCT and other US-based CHW programs.

Interviewees across constituent groups identified a number of barriers impeding implementation that, though focused specifically on the IMPaCT model, may be generalizable to other CHW programs, including challenges with clinical integration and financial barriers. Despite the focus on clinical integration within the IMPaCT model, participants noted that the value of CHWs was often underrecognized within health systems. Consistent with previous literature,^28,29^ the CHW role was often misunderstood by other health care professionals, leading CHWs to feel disrespected. Also consistent with previous literature,^30,31^ interviewees highlighted challenges associated with finances, including program costs (inclusive of overall operational costs of running a CHW program combined with specific IMPaCT-related costs) and lack of secure funding, making program sustainment precarious and, at times, leading to challenges with fidelity, even in the face of strong return on investment associated with the program.

Despite implementation barriers, participants highlighted several facilitators, 2 of which were emphasized. First, interviewees noted how the IMPaCT model prioritized and enhanced relationships, including those between the health system leaders and the IMPaCT implementation team (eg, during training and consultation), within the health system’s IMPaCT delivery team (eg, between CHWs and their managers, among CHWs), and between CHWs and patients (eg, the IMPaCT model is longer and involves more in-depth contact between CHWs and patients than some other programs), thus overcoming some of the barriers to health experienced by marginalized communities.^32,33,34^ While IMPaCT’s emphasis on longitudinal, patient-centered support is not unique among CHW models, it does distinguish IMPaCT from other social determinants of health–focused interventions led by CHWs, such as screen-and-refer models that prioritize quick connection to resources and models focused on health education and adherence to specific diagnostic procedures or treatments (eg, cancer screening, appointment follow-up, medication adherence). Some interviewees highlighted that the emphasis on in-depth relationships within the IMPaCT model sets it apart from other CHW programs and contributes to high levels of satisfaction among staff in their roles and patients with the services provided. This emphasis on relationship building is consistent with the broader literature, suggesting that an emphasis on rapport building and relationships may facilitate patient engagement.^35^ Second, participants highlighted IMPaCT’s program components and implementation approach, consistent with best practices in the field,^36,37^ as robust facilitators of success.

Across all 5 domains and from the perspectives of all constituent groups, a number of burdens within the CHW workforce were identified, many of which may be associated with other CHW programs. First, CHWs are individuals who are selected for their personal characteristics, including empathy, passion, and a desire to help others.^38^ Given these qualities, CHWs may be prone to taking on additional responsibilities requested by program staff and patients, especially if they perceive that health systems are unable to meet patients’ needs.^39^ Second, in the context of the COVID-19 pandemic, CHWs faced changing work responsibilities, diminished boundaries with patients, funding uncertainty, and exposure to situations for which they felt they had insufficient training. Since many CHWs share minoritized and marginalized identities with their patients, they are often affected by the same social determinants of health, leaving them vulnerable to additional stressors.^40^ On top of these challenges, CHWs were often met with clinical integration barriers within health systems, where they felt misunderstood, unwelcome, and undermined. Finally, an additional challenge specific to IMPaCT was the need to tailor the materials to the target patient population, a task that at times fell on CHWs given their deep cultural understanding and language skills. These barriers may be associated with burnout and the high turnover rate of CHWs (Table 4).^41^ There is growing literature on the importance of attending to CHW workforce needs, though much of this work has taken place outside the US.^42^ Given the very limited literature on burnout among US-based CHWs, more work is needed.^43,44,45,46^ Notably, health care professional burnout is a broader issue within the US, as highlighted in a recent advisory by the Office of the Surgeon General.^47^

These findings can be leveraged to inform meaningful future research and policy work to support CHWs and maximize their contributions to public health. For example, despite IMPaCT’s emphasis on clinical integration, both program leadership and CHWs identified substantial challenges with integration. Strategies to mitigate this barrier may involve providing formalized education to other health care professionals (eg, physicians, social workers, and nurses) about the CHW role, creating standardized guides on how best to collaborate with CHWs, and developing policies for integration at the organizational level.^48,49,50^ These results also point to challenges with sustainable program financing. While there is no quick fix for structural and systemic barriers within the outer setting, including the financial barriers identified, the salience of this barrier within our study and the broader literature underscores the need for continued advocacy and policy work to make CHW programs financially sustainable, such as through Medicaid and Medicare reimbursement.^51^ A recent Centers for Medicare & Medicaid Services rule allowing for Medicare reimbursement for services provided by CHWs, effective January 1, 2024, may help to mitigate this barrier.^52^ Future work should examine perspectives of individuals across the implementation and delivery spectrum regarding the extent to which this ruling may ameliorate financial barriers.

### Limitations

Our study has several limitations. First, selection bias may have resulted in differences between leaders, managers, CHWs, and patients who participated in interviews and those who did not. For example, patients whose contact information was provided by CHWs may have been more engaged or satisfied with the program than the average patient. Second, since the implementation evaluation team was affiliated with the same institution as the IMPaCT implementation team, social desirability biases may have influenced the results. Finally, this study took place during the first 15 months of the COVID-19 pandemic, which may make our findings less generalizable to a nonpandemic context.

## Conclusions

This study captures perspectives from individuals across multiple geographically diverse health systems about the implementation and delivery of the IMPaCT CHW model. While these findings are specifically based on implementation of 1 evidence-based CHW model, the barriers and facilitators identified may be relevant to CHW models more broadly. Our findings point to the importance of the emphasis on relationships within IMPaCT, as well as of the structured, patient-centered program design. They also point to potential threats to sustaining IMPaCT and similar programs, including clinical integration challenges, financial barriers, and burdens on the CHW workforce that may lead to burnout over time. Given that CHWs cannot transform health systems on their own, larger system-level change is needed to address extant challenges and prevent the identified barriers from undermining the potential contribution and reach of the CHW workforce.

## Notes

Supplement 1. **eMethods 1.** Interview Guides **eMethods 2.** Detailed Description of Themes **eMethods 3.** Additional Individualized Management of Person-Centered Targets (IMPaCT) Quotes **eFigure.** Frequently Used Descriptors of Community Health Workers

Supplement 2.
Data Sharing Statement
