# Supplementary material for: Barriers and Facilitators to Implementing an Evidence-Based Community Health Worker Model
Source: JAMA Health Forum. 2024 Mar 8;5(3):e240034. doi: 10.1001/jamahealthforum.2024.0034 (PMC10924240; doi:10.1001/jamahealthforum.2024.0034)
Supplement: Supplement 1. — eMethods 1. Interview Guides eMethods 2. Detailed Description of Themes eMethods 3. Additional Individualized Management of Person-Centered Targets (IMPaCT) Quotes eFigure. Frequently Used Descriptors of Community Health Workers [file jamahealthforum-e240034-s001.pdf]

Supplemental Online Content

Schriger SH, Knowles M, Daglieri T, Kangovi S, Beidas RS. Barriers and facilitators to implementing an evidence-based community health worker model. *JAMA Health Forum*. 2024;5(3):e240034. doi:10.1001/jamahealthforum.2024.0034

eMethods 1. Interview Guides

eMethods 2. Detailed Description of Themes

eMethods 3. Additional Individualized Management of Person-Centered Targets (IMPaCT) Quotes

eFigure. Frequently Used Descriptors of Community Health Workers

This supplemental material has been provided by the authors to give readers additional information about their work.

© 2024 Schriger SH et al. *JAMA Health Forum.*

eMethods 1. Interview Guides

Leadership Interview Guide

We are interested in understanding your thoughts about the IMPaCT community health worker program. We would like to hear your perspective on the fit, relevance, and compatibility of IMPaCT in your health system and what it was like to launch, implement, and sustain the program. We are particularly interested in your thoughts about how easy it was to put it in place, what might keep or stop your organization from using it, and what its advantages and disadvantages might be. It is important for you to know that there are no right answers; we are most interested in your opinion, as an expert in what you do.

Q (Participant information): First, could you tell me your job title and briefly what you do in this role? How would you describe your involvement in standing up your community health worker program?

Q (role of CHWs): I would like to hear your thoughts on the role of community health workers in healthcare organizations with regard to improving population health.

Q (General implementation process): Walk me through the experience of implementing IMPaCT from the beginning and your general reflections on the process.

> As a reminder, the Penn IMPaCT implementation bundle consists of a set of specific activities. I will now walk you through each stage and ask you to reflect on each stage.
>
> The first step was stakeholder engagement, where members of the Penn IMPaCT team did a site visit to your organization. What did you think of this stage? What made this easy or difficult for your organization?
>
> The second step was strategic planning, where the Penn IMPaCT team used information from the site visit and independent research to develop and refine a Blueprint for your CHW program. What did you think of this stage? What made this easy or difficult for your organization?
>
> The third step was partnering to support your use of HOMEBASE, IMPaCT's CHW-specific application, or another existing documentation and reporting software. What did you think of this stage? What made this easy or difficult for your organization? If you decided not to use HOMEBASE, what were some of the factors that went into that decision?
>
> The fourth step was tailoring IMPaCT's intervention materials (CHW, manager, and director manuals) based on local needs and preferences. What did you think of this stage? What made this easy or difficult for your organization?
>
> The fifth step was recruiting and hiring your team members, which included CHWs, managers, directors and coordinators. What did you think of this stage? What made this easy or difficult for your organization?
>
> The sixth step was partnering to provide comprehensive, customized training for your team. This included in-person or remote CHW training, manager and director training on-site in Philadelphia, and may have also included coordinator training. What did you think of this stage? What made this easy or difficult for your organization?
>
> The seventh step was to provide ongoing support and troubleshooting after you started enrolling patients. What did you think of this stage? What made this easy or difficult for your organization?

Q (COVID-19 and structural racism): How have recent events related to COVID-19 and structural racism affected the role of CHWs in your organization?

© 2024 Schriger SH et al. *JAMA Health Forum.*

Q (Barriers): Thinking across each of these stages, what were the biggest barriers to implementing your CHW program? By barriers, we mean anything that got in the way of launching, implementing or sustaining (if at that point) it in your setting.

Make sure to probe for barriers related to CFIR domains: intervention characteristics, individuals involved, inner setting, and outer setting.

Q (Facilitators): Thinking across each of these stages, what were the biggest facilitators to implementing IMPaCT? By facilitators, we mean anything that made it easier to launch, implement or sustain (if at that point) it in your setting.

Make sure to probe for facilitators related to CFIR domains: intervention characteristics, individuals involved, inner setting, and outer setting.

Q (Intervention characteristics): *Relative advantage*. What, if any, other CHW programs, or programs to help patients living in poverty, are currently in place where you work? How does IMPaCT compare to these existing programs (advantages, disadvantages)? *Adaptability.* What kinds of changes or alterations to IMPaCT have you had to make in order to make it a good match for where you work in order for it to work effectively? What if any other changes or alterations would be helpful? *Cost.* Knowing what you know about the costs of implementing IMPaCT were these costs prohibitive or limiting in any way?

Q (Inner Setting): Tell me about how your healthcare organization or clinical setting affected launching and implementing IMPaCT? Were there any infrastructure and operational procedures or policies that were needed for implementation? What resources or support were needed to effectively implement your community health worker program in your setting?

Q (Outer Setting): Tell me about how your larger context (i.e., local, state, or national-level) affected launching and implementing your community health worker program? Were there any policies, regulations, and/or laws that were relevant to implementation?

Q (Individuals involved): Now I will be asking you about the people who are responsible for launching, implementing, and sustaining your community health worker program. *Adopters.* Who, specifically, in your health system made the decision to implement this intervention? How did they support implementation? Was there anyone who should have been involved in adoption that was not? *Implementers*. Who, specifically, in your health system is involved in the implementation of your community health worker program? Let's start by listing all of the potential people (doctors, nurses, medical assistants, CHWs, managers, directors, coordinators) who might be involved and what their roles are in the delivery of your community health worker program. Was there anyone who should have been involved in implementation who was not? *Sustainment.* Who is responsible for ensuring that your community health worker program is implemented correctly and consistently over time? How would you know that your community health worker program has been sustained? Is there anyone who should have been involved in sustainment that was not?

Q (Anything else): Is there anything else you'd like to add before we conclude with the interview?

© 2024 Schriger SH et al. *JAMA Health Forum.*

Manager Interview Guide

We are interested in speaking with you about the IMPaCT community health worker program. We would like to hear your perspective as a manager about how IMPaCT fits in your health system and what it was like to use the program. We are particularly interested in your thoughts about how easy or challenging it was to put it in place, what the advantages and disadvantages might be of using it, and what it was like to manage CHWs. It is important for you to know that there are no right answers; we are most interested in your opinion, as an expert in what you do.

Q (Participant information): First, could you tell me your job title and briefly what you do in this role? How would you describe your relationship to IMPaCT?

> *Can probe for various aspects of their training (including 3-days of data-driven performance management, burnout prevention and safety protocols training) and/or their responsibilities (including providing real-time support, ongoing training, performance assessment, and helping with clinical integration)*

Q (role of CHWs): Tell me about your perspective on the role of CHWs as part of a healthcare delivery system. What role do they play in your organization specifically? Can you tell me about the benefits and also some of the hard parts of including CHWs?

Q (General implementation process): When did you first get involved in the implementation of the IMPaCT program?

> Members of the IMPaCT team did a site visit to your organization. Were you there for that meeting? If so, what did you think of it? What made the meeting easy or difficult for your organization?
>
> The IMPaCT team used information from the site visit and independent research to develop and refine a Blueprint for your CHW program. Have you seen that document? If so, what did you think of the Blueprint?
>
> Does your organization use HOMEBASE? If so, what has made it easy or difficult for your organization to use?
>
> IMPaCT's materials (CHW, manager, and director manuals) are adapted, in some cases, based on local needs and preferences. Were you involved in adapting IMPaCT materials for your health system? If so, what did you think of this process? What made it easy or difficult for your organization?
>
> The IMPaCT team often supports organizations through the process of recruiting and hiring team members, which may have included CHWs, managers, directors and coordinators. Were you involved in that process? What did you think of the hiring process? What made it easy or difficult for your organization?
>
> The IMPaCT team partnered with your organization to provide comprehensive, customized training for your team. This included in-person or remote CHW training, manager and director training on-site in Philadelphia, and may have also included coordinator training. What did you think of the training? What made this easy or difficult for your organization?
>
> The IMPaCT team provided ongoing support and troubleshooting after you started enrolling patients. What did or do you think of the support you received? What made this easy or difficult for your organization? Now that you have had some experience working as a manager, are there any tools, training or other forms of support that you are particularly glad that you received? Are there any tools, training, or other forms of support that you wish you had gotten and think would have been useful?
>
> Can you tell me a bit about your experience with clinical integration. How has that process gone for you What has made it easy and what has made it challenging?

© 2024 Schriger SH et al. *JAMA Health Forum.*

Q (Barriers): Thinking across all of your team's work with the IMPaCT team, what were the biggest barriers to rolling out and/or using IMPaCT? By barriers, we mean anything that got in the way of rolling out, using, or sustaining (if at that point) it in your setting.

Make sure to probe for barriers related to CFIR domains: intervention characteristics, individuals involved, inner setting, and outer setting.

Q (Facilitators): Thinking across all of your team's work with the IMPaCT team, what were the biggest facilitators to rolling out and/or using IMPaCT? By facilitators, we mean anything that made it easier to roll out, use, or sustain (if at that point) it in your setting.

Make sure to probe for facilitators related to CFIR domains: intervention characteristics, individuals involved, inner setting, and outer setting.

Q (Intervention characteristics): *Relative advantage*. What, if any, other CHW programs, or programs to help patients living in poverty, are currently in place where you work? How does IMPaCT compare to these existing programs (advantages, disadvantages)? *Adaptability.* What kinds of changes or alterations to IMPaCT are needed to make it a good match for where you work in order for it to work effectively?

Q (Inner Setting): Tell me about how your specific healthcare organization or clinical setting affected rolling out and using IMPaCT and/or managing CHWs? Were there any infrastructure and operational procedures or policies that were needed for you to effectively manage the CHWs? What resources or support were needed to effectively roll out/use the program and/or manage CHWs?

Q (Outer Setting): Tell me about how your larger context (i.e., local, state, or national-level) affected rolling out and using IMPaCT? Were there any policies, regulations, and/or laws that were relevant to rolling out/using the program and/or managing CHWs?

Q (COVID-19 and structural racism): How has COVID-19 affected your work as a manager? How has your role changed since the start of the pandemic? Have you been able to find alternative ways of managing CHWs during this time, or has your work mainly been put on hold for the moment? Is there anything that you have learned during this time that you think you will take forward? How has structural racism and recent events in the community affected your work?

Q (Anything else): Is there anything else you'd like to add before we conclude with the interview?

© 2024 Schriger SH et al. *JAMA Health Forum.*

CHW Interview Guide

We are interested in speaking with you about your experience as a community health worker at [health system]. We are particularly interested in your thoughts about how easy or challenging it has been to carry out your role, what the advantages and disadvantages are to the CHW model that your organization is using (which is the IMPaCT model developed at UPenn in Philadelphia) and what it has been like to work with patients. It is important for you to know that there are no right answers; we are most interested in your opinion, as an expert in what you do.

Q (Participant information): Tell me about your job and what you do in your role as an IMPaCT CHW. What types of needs do you see most often in the patients you work with? Which ones are easiest to address? Which are hardest to address?

> *Can probe for patient needs such as emotional/social support, advocacy, navigation, resources, helping them make changes*

Q (Role of CHWs): Thank you for sharing more about what you do. Now I am going to ask you more about the role of CHWs in general. From your perspective, what role do CHWs play in your organization and in the community? Can you tell me about the benefits and also some of the hard parts of being a CHW? How connected do you feel you and other CHWs are to the communities you work with?

Q (Roles and responsibilities): At your organization, how clearly defined are your roles and responsibilities as a CHW? Is your workload manageable? What is your relationship like with other CHWs?

> *Can probe for any problems caused by lack of clarity of role, peer support*

Q (Retention and career path): What keeps you in your role as a CHW? Is there anything that would cause you to want to leave or that would get in the way of you continuing? Do you have ideas about how to keep CHWs wanting to stay in their roles long-term? How do you envision your career path moving forward? Do you view working as a CHW as a long-term position? What would you want your future work to look like?

Q (Supervision): How does your manager support you? How often do you meet, and what do you meet about? Do you feel you get enough ongoing support and training?

Q (General implementation process): Now we are going to ask a bit about your experience working as a CHW in a system implementing the IMPaCT model. We have a series of questions about specific steps in this process that you may have been involved in.

> The IMPaCT team often supports organizations through the process of recruiting and hiring team members, which in addition to directors and managers, may have also included you and other CHWs. Can you tell me a bit about the hiring process? What did you think of it? What made it easy or difficult? What do you think a program would need to do in order to attract and recruit the best CHWs from your community
>
> > *May want to probe for whether they were hired before or after the IMPaCT model was implemented (i.e., whether the hiring practices used were in line with the IMPaCT model)*
>
> The IMPaCT team partnered with your organization to provide comprehensive, customized training for your team. This included in-person training in Philadelphia for some team members, and either in-person or remote CHW training. What did you think of the training? What made this easy or difficult for you or your coworkers? Now that you have had some experience working as a CHW, are there any tools, training or other forms of support that you are particularly glad that you received? Are there any tools, training, or other forms of support that you wish you had gotten and think would have been useful?
>
> The IMPaCT team provided ongoing support and troubleshooting after you started enrolling patients, which you may or may not have been involved with. Have you had any interaction with the IMPaCT team? If so,

what did or do you think of the support you received? What made this easy or difficult for you or your organization?

Can you tell me a bit about your experience getting integrated with the patient care team? How has that process gone for you? What is your relationship with doctors, nurses, social workers, etc. like? What has made it easy and what has made it challenging for you to work with them on patient care?

Tell me a bit about how you carry out documentation of your work with patients. Does your organization use HOMEBASE or a different software? What has made it easy or difficult to carry out documentation? How has the documentation system you use made things easier or harder to keep track of patient information and carry out your work with patients?

Tell me about the manuals that you use in your work. How are these manuals helpful and/or unhelpful? In what ways do they fit (or not fit) for the people and places that are specific to your work. What suggestions do you have for improving them?

Q (Ending relationship): Have you had to end your relationship with any patients? If so, how was this process? What made it difficult or easy to do this? Have you been in touch with any patients since your official work with them ended?

Q (Mental health): Did the topic of mental health come up during your work with your patients? If yes, what kinds of mental health-related goals did you work on with them? Do you think the IMPaCT program address mental health concerns as effectively as it addresses physical health concerns? Do you have anything else to add about the topic of mental health in relation to this program?

Q (COVID-19): How has COVID-19 affected your work as a CHW? How has your role and you responsibilities changed since the start of the pandemic? Have you been able to find alternative ways of carrying out your job? What was done that made you feel supported? What could have been done to make you feel more supported? Is there anything that you have learned during this time that you think you will take forward even after the pandemic?

Q (Racism) How have recent national conversations about structural and day-to-day racism and recent events in the community affected your work?

Q (Barriers): Thinking across all aspects of your work as a CHW and your involvement with your team, what are the biggest barriers? By barriers, we mean anything that got in the way of applying, joining the team, and/or carrying out your roles and responsibilities as a CHW. This could include getting onboarded into this role, carrying out your daily responsibilities, or anything else that is challenging or was challenging previously. What do you think would get in the way of CHWs remaining in this position for a long time?

Q (Facilitators): Thinking across all aspects of your work as a CHW and your involvement with the team, what are the biggest facilitators? By facilitators, we mean anything that made it easier to apply, join the team, and/or carry out your roles and responsibilities as a CHW. This could include getting onboarded into this role, carrying out your day to day responsibilities, or anything else that made it easy and/or continues to make it easy. What factors do you think would make it most likely to stay in this role for the long-term?

Q (Sustainment): Thinking about your CHW program as a whole, what are your thoughts on sustaining it over the long term? Are there any changes you think would need to be me made to keep this program running for a long time?

Q (Intervention characteristics): *Relative advantage*. Have you been employed as a CHW in other programs or in your current setting before they began using the IMPaCT model? How does IMPaCT compare to those programs (advantages, disadvantages) and/or how did it make things better/worse than before? *Adaptability.* What kinds of

© 2024 Schriger SH et al. *JAMA Health Forum.*

changes or alterations to the IMPaCT model are needed to make it a good match for where you work? Do you have any suggestions for improvement?

Q (Inner Setting): Tell me about how your specific healthcare organization or clinical setting affects your daily work as a CHW. What policies, resources, or support were needed in order for you to effectively do your job?

Can probe for aspects of the inner setting such as organizational climate and culture

Q (Outer Setting): Tell me about how your larger context (i.e., local, state, or national-level) affects your work as a CHW. Are there any policies, regulations, and/or laws that have affected your work?

Q (Anything else): Is there anything else you'd like to add before we conclude with the interview?

© 2024 Schriger SH et al. *JAMA Health Forum.*

Patient Interview Guide

We are interested in talking with you about your experience working with your community health worker (which I will refer to as CHW). We are interested in hearing about how easy or hard it was to work with your CHW, what the advantages and disadvantages might be of working with your CHW, and any other thoughts you have about your experience. It is important for you to know that there are no right answers and that what you tell us will not affect your care in any way or be shared with your CHW; we are just interested in hearing your opinion.

Q (Opening question about experience working with CHW): Tell me about your general experience working with your CHW. What was it like working with your CHW and what was your relationship like? What did you like about it or what was good about it? What didn't you like about it or what was bad about it? What were the 1-2 main things you worked on?

Q (Initial impression and deterrents): What did you think at first when you first learned that you could work with a CHW?

> *Probes: What thoughts, concerns, questions, doubts, or reservations did you have when you first heard about the program?*

Q (Definition of CHW and description of specific work with CHW): How would you describe the work of a CHW to someone else? How did you and your CHW decide what to work on together, and how long did you work together?

> *Probe across the relationship with CHW: When did you get connected with your CHW and how did you meet? What was your understanding of what you would be working on together? What were your first impressions of your CHW and how did those impressions change over time? How long have you been working together, and are you still working together?*

Q (Reflections on experience): Now I will ask you to reflect a bit more on your experience working with your CHW

> **Q (Length of relationship and communication):** Thinking back on your whole experience working with your CHW, what do you think of the length of time that you worked together (too short/too long) and about the degree of communication (too much/too little)?
>
> **Q (Success and challenge):** Tell me about something that you and your CHW did that really made a difference in your life. How did you decide that was something to work on? What did your CHW do to help achieve this and what id you do? How do you feel about this success? Now tell me about a goal that you set with your CHW that you were not able to accomplish. How did you decide that was something you wanted to work on? What got in the way of you meeting your goal? What could you have done differently to help accomplish this goal and what could your CHW have done differently?

Q (Final impression): Now that you have some experience working with your CHW, what is your confidence in your CHW's knowledge and skills? How well connected did you feel your CHW was to your community? How much did they understand your community? Did your CHW help you to make changes in your behavior, and have you maintained these changes?

Q (Characteristics of CHWs): Thinking about CHWs in general, what personal qualities of a CHW (e.g. personality) do you think would make it easier to work with them? Are there any qualities that you can think of that would make it harder to work with a CHW?

Q (Clinical integration): Can you tell me a bit about your experience with how your CHW related to the rest of your care team? Was your CHW able to work with your other health care providers like doctors and nurses or was this hard?

© 2024 Schriger SH et al. *JAMA Health Forum.*

Q (Ending relationship – for those who are no longer working with their CHW) Can you tell me a bit about what it was like to end your relationship with your CHW? What made it easy or difficult? Have you spoken with your CHW since that time?

Q (Mental health) Did the topic of mental health come up during your work with your CHW? If yes, what did you work on? In what ways, if any, has working with your CHW affected your mental health? Did the CHW program address your mental health concerns as effectively as it addressed your physical health concerns?
Do you have anything else to add about the topic of mental health in relation to this program?

Q (COVID-19): Were you working with your CHW before the COVID-19 pandemic? How has COVID-19 changed your relationship with your CHW and the work you did together? Did the pandemic make working with your CHW more difficult in any way? Did the pandemic make working with your CHW easier in any way? What did your CHW do during the pandemic that made you feel supported, and what could your CHW have done to make you feel more supported?

Q (Racism) How have recent national conversations about structural and day-to-day racism and recent events in the community affected your work with your CHW?

Q (Barriers): Thinking about all of the times that you worked with your CHW, what were the biggest challenges? This could mean things that got in the way of working together, things that made it difficult to meet up or talk, or things that made it difficult to complete the goals that you set with your CHW. What aspects of working with your CHW were the most difficult, and what are areas do you think could be improved upon?

Q (Facilitators): Thinking about your time working with your CHW, what were the biggest successes? How is your life different or better? What did you like most about working with your CHW – in other words, what would you recommend keeping the same for other patients in the future?

Q (Intervention characteristics): Have you worked with a CHW or someone like a CHW previously? How did working with your CHW compare to those previous experiences? Do you have any feedback about how to improve the services provided to you from your CHW?

Q (Anything else): Is there anything else you'd like to add before we conclude with the interview?

© 2024 Schriger SH et al. *JAMA Health Forum.*

eMethods 2. Detailed Description of Themes

Innovation

Program cost (barrier)

The cost of IMPaCT (both upfront and ongoing certification costs) was cited as a barrier to its use. Participants also noted that the cost of the recommended documentation software was prohibitive. Program cost influenced decisions about program renewal as sites had concerns about ability to continue paying for IMPaCT.

Applicability to specific marginalized populations (barrier)

Despite generally high levels of acceptability of the IMPaCT program, participants frequently commented that IMPaCT materials lacked applicability to specific marginalized populations, particularly undocumented and non-English-speaking patients. Interviewees noted that the manuals, while flexible, often needed significant adaptation to fit their patient population, including more culturally-relevant examples to reflect diverse culture, literacy status, rural location, immigration status, and language. At times, CHWs were called upon to contribute to adaptations given their unique skillset and familiarity with patient populations (both in terms of literal language translation and broader cultural brokerage). Participants suggested that IMPaCT could be strengthened by creating a repository of adapted materials from participating sites so that over time, the IMPaCT team can better support each new organization with the needs of their unique patient population.

Evidence base of program (facilitator)

Participants (particularly health system leaders and managers) highlighted the robust evidence-base supporting IMPaCT's effectiveness as a strong facilitator of adoption and implementation; it led to a quicker and greater sense of trust in the IMPaCT team and their recommendations.

Program design (facilitator)

The IMPaCT program design was lauded for its clear organization, highly structured workflow, straightforward protocols, defined roles and responsibilities, and structured tools, such as the specialized hiring recommendations and the Meet the Patient Interview (a semi-structured initial assessment tool). Participants noted that these tools gave IMPaCT an advantage over other CHW programs. For example, interviewees cited that the unique community-based hiring recommendations (hiring based on personal characteristics rather than specific work experience) led to IMPaCT CHWs with standout qualities including empathy, trustworthiness, and passion. Participants also lauded the program's comprehensiveness in terms of its length (i.e., several months) and depth (i.e., high level of contact with patients); flexibility and adaptability (i.e., disease-agnostic design in which patients get to choose what to work on); and holistic (i.e., more than just medical needs) and empowerment-based approach. Further, participants identified benefits of implementation strategies built into the program, highlighting continuing education opportunities (such as learning collaboratives for CHWs and program leaders across IMPaCT implementation sites nationally that facilitated mutual learning and community-building).

Outer setting

Economic and political climate (barrier)

Participants cited the broader economic climate and legislation affecting CHW programs and patients as a barrier to implementation. Interviewees noted that budget cuts threatened program sustainability, and general economic hardships meant that patients were often facing extreme poverty and homelessness, resulting in higher social needs from the program. Interviewees noted that lack of support from larger state bodies or external funding sources makes program sustainment difficult, as do policies affecting CHW programs (e.g., limited reimbursement by

© 2024 Schriger SH et al. *JAMA Health Forum.*

Medicaid/Medicare, reliance on grant funding, etc.). Further, federal changes in immigration and documentation policy (e.g., public charge rules, Trump-era immigration policies), created additional challenges for undocumented patients and created fear and hesitation for this population in utilizing programs and services.

COVID-19 pandemic (barrier and facilitator)

The COVID-19 pandemic was cited as both a barrier and facilitator to implementation. In some ways, the pandemic exacerbated existing challenges, such as existing financial strains within health systems and patient economic hardships. It also widened disparities for populations, such as undocumented patients (who did not receive stimulus checks). The pandemic also created a host of new challenges related to the shift to a virtual format, such as online rather than in-person trainings, which some interviewees felt were unideal. Further, some CHWs were asked to fulfill novel tasks that made them feel uncomfortable, such as home visits. Participants also identified pandemic-related facilitators, including a greater sense of team comradery, and expansion of patient services (e.g., additional forms of patient support) and modalities (e.g., option for use of virtual platforms). Several participants noted that the virtual format became acceptable when done in conjunction with at least some in-person contact.

Inner setting

Clinical integration (barrier)

Clinical integration, or the extent to which CHWs are able to collaborate with other health care workers, was identified as a significant challenge by leaders, managers, and CHWs. Despite emphasis on clinical integration within the model, across all sites, interviewees detailed difficulties with integrating CHWs into care settings due to confusion about or lack of respect for their role from nurses, social workers, and other allied health professionals. CHWs reported feeling misunderstood and in some cases even undermined or "blocked" by other health professionals. CHWs were often met with skepticism due to their lack of "credibility" (i.e., no formal clinical education or licensure) and even myths and distrust (e.g., the belief that CHWs will inappropriately discourage patients from coming to the hospital). CHWs expressed frustration at having to constantly re-introduce themselves to other health professionals who they had met previously. Participants noted several factors associated with more successful clinical integration, including CHWs who were internal hires (i.e., already working at the organization) as well as supportive physicians. Unlike program staff, patients described positive impressions of integration, perceiving their care team as a united front.

Individuals

Leadership at organization (barrier and facilitator)

Participants identified the significant role of leaders at an organization in influencing implementation success and the relative priority of the program within their organization. Interviewees noted several challenges with executive-buy in, particularly for leaders who are not as research- or data-focused. Unsupportive leadership was cited as a threat to program sustainability. When leadership were supportive, this was viewed as a large facilitator of program success.

Characteristics of CHWs (facilitator)

The personal characteristics of CHWs were unanimously cited as a facilitator of program implementation. Participants identified this workforce as the future of population health, describing them as indispensable to health systems. CHWs were perceived across the board as going above and beyond, and patients cited their ability to form genuine connections as extremely therapeutic and impactful. Respondents highlighted CHWs' reliability, empathy, loyalty, trustworthiness, knowledge, and personability, among other qualities.

© 2024 Schriger SH et al. *JAMA Health Forum.*

IMPaCT implementation team (facilitator)

Many participants identified the IMPaCT implementation team as a strong implementation facilitator, describing them as engaging and charismatic. Participants particularly appreciated the IMPaCT team trainers' enthusiasm and content knowledge and the team's extensive real-world experience (vs. purely academic or theoretical knowledge).

Implementation process

Program boundaries (barrier)

Participants commented on the difficulty CHWs had maintaining program boundaries. Nearly all CHWs described that despite an "official" program end date, a subset of patients would continue to contact them beyond this date to share updates, make requests, or simply remain in touch. CHWs explained that many patients expressed feeling more safety and trust in their relationship with their CHW than with other providers, leading to a dependence on CHWs and a resistance to referrals to another provider (e.g., a mental health clinician). As a result of their unique role, CHWs described feeling reluctant to take time off, especially if they were the only team members who spoke a patient's language or who could perform a certain duty.

Training (barrier and facilitator)

By and large, participants cited the IMPaCT training as an implementation facilitator. Participants highlighted the hands-on, active strategies used in trainings, such as role-playing. Several interviewees expressed appreciation for a CHW training conducted entirely in Spanish. Participants also noted several areas for improvement, including more content related to crisis management, domestic violence, and mental health. Several CHWs described feeling poorly equipped to handle situations in these domains, and some CHWs noted the negative impact on their own mental health (e.g., feeling overwhelmed due to hearing details of traumatic events in patients' lives). Participants suggested that further training in mental health could allow CHWs to feel more equipped to respond to situations involving mental health-related concerns, such as better distinguishing patients appropriate for mental health referrals from those simply expressing emotions during a difficult time.

Fidelity (barrier and facilitator)

At times, conflicts arose between core IMPaCT recommendations and system constraints (due to financial or procedural issues). For example, IMPaCT's recommended hiring and recruitment process, though viewed favorably by participants, were often prohibited by human resources policies. Similarly, sites were unable to adopt recommended documentation software due to cost and infrastructure issues. This was frustrating to many interviewees, as the proprietary software recommended by IMPaCT was viewed as highly acceptable and preferable. Despite the desire to adopt all program components, such constraints led to decreased fidelity to the model. Across the board, participants agreed that the IMPaCT recommendations, when followed, were helpful, and that when fidelity was possible, it facilitated the success of their program.

© 2024 Schriger SH et al. *JAMA Health Forum.*

eMethods 3. Additional Individualized Management of Person-Centered Targets (IMPaCT) Quotes

Innovation

Program cost (barrier)

“It’s pricy and hard to sustain over a long period of time.” -Leader 2, Site 5

(About not being able to purchase IMPaCT recommended software): “I think it would have definitely been more helpful to just use the tool that they already offered. But I know funding sometimes isn’t there.” – Manager, Site 3

(About not being able to purchase IMPaCT recommended software): “I mean it looked amazing. But it came down to cost. We were already going to be spending so much on training, including travel cost for the managers and myself at the time. So it really came down to cost, and also I was concerned that even if we were able to afford it for one year, would we be able to sustain that cost for the years to come?” – Leader 2, Site 5

Applicability to specific marginalized populations (barrier)

I remember the Spanish versions being difficult to read. Like, somebody tried to translate the English to the Spanish and it didn’t really flow very well. — CHW 1, Site 5

“I know that it’s difficult to do that when you’re working for different programs and different organizations, but I think that would probably be my suggestion; just making sure that you have the materials for the different populations.” – Manager, Site 1

Evidence base of program (facilitator)

“It’s so nice to be able to build a data-driven program, versus just like going in the dark with all these interventions.” -Leader 1, Site 1

“Everybody is very excited that we’ve brought an evidence-based model into the area. I think people have tried programs, they might not have called them community health workers, but they have tried similar programs, but there’s never been anything that had evidence that they were actually making significant progress. – Manager, Site 2

“My ability to say to HR, “Hey, we’re actually doing this for a very specific reason, it’s because of this model like here, look,” was the only way that I think I would be able to do it. They still didn’t believe that it would work; so I think that was the most concrete example. The fact that having the materials, having the evidence base behind it, comes in handy at all different points in the process where you wouldn’t necessarily think you’d need it, and then all of a sudden I’m like, “Hold on a second. Let me pull it out, let me show you, this is why we’re doing this. – Leader 2, Site 1

Program design (facilitator)

I just really love this model and I’m really grateful that I was trained under this model because I used to work as a CHW back in the day, and I used to oversee CHWs… really didn’t have no model. So this was more of a structure, I’m able to provide my staff the real support they need. I love overall the model, the way it helps you help not only my staff but my patients and the community overall. – Manager, Site 5

(About other CHW programs at hospital): “They’re just getting the patient for like three days, they don’t really know the patient. We’re getting the patient for longer than that, and we’re getting to know the patient. So it looks different… we had like a shared space with all the CHWs from other programs, and it was way different from ours – CHW 1, Site 1

© 2024 Schriger SH et al. *JAMA Health Forum.*

Outer setting

Economic and political climate (barrier)

“There’s a phrase going around, “No money, no mission.” That’s one of the struggles that we’re having.” -Leader 1, Site 5

Working with the undocumented, and then trying to find resources for them and providing that support they needed, especially when the pandemic began they weren’t getting no stimulus checks, they didn’t have no income coming in and just really trying to figure out how are we gonna help this patient get food this week? How are we gonna help this patient feel secure enough to… yeah, it was a hard time with our undocumented population, for sure – Manager, Site 5

COVID-19 pandemic (barrier and facilitator)

It’s harder to kinda connect with patients over the phone. We’re so used to going to home visits, being at the doctor, being at the bedside when they’re getting discharged, so it’s a little bit harder to connect because I’m just a phone person now. They don’t see who I am.” -CHW 1, Site 1

“It definitely made a difference, because I feel like now we’re closer, during the pandemic we got a little closer. Where before we were kind of like stick to our schedule. So I think that has been a benefit.” -Manager, Site 3

“I feel like I was able to get people to go to their appointments a lot better when I was meeting them there [in person].” -CHW 1, Site 2

“The process of how to refer patients to us kind of makes it difficult when everything is remote and their time is very limited.” -Manager, Site 5

Inner setting

Clinical integration (barrier)

“We’re looked at as not important in the role that we play.” -CHW 1, Site 5

The struggle or the difficulties that we have found, or that I have found, with CHWs being integrated is mainly, because it’s a new position, is kind of getting the respect or the acknowledgement of their position. So for example… they’re kind of like, “what did you go to school for?” And “what kind of clinical background do you have?” – Manager, Site 3

“Sometimes they feel like we’re stepping on their toes, or they feel like we’re trying to take their positions. So we really experienced that a lot when we first got there. They weren’t as welcoming as I thought they should be, seeing what we’re trying to do for the patients. That was a little challenging because every time I walk in a room, after I talked to some of these doctors and nurses already, when I walk in a new room they always want me to reintroduce myself, or retell them what the program is. That was a little bit challenging for me because I’m walking in with patients sometimes, and they see that I’m getting questioned. So it’s like, “Do you really work here?” You know, I didn’t like that, because I felt like I don’t want this patient to think that I’m just telling a story, I want them to know that I’m here for them. I’m here to help them, and I have the foundation to do that. So when it comes down to the hospital staff not being supportive, it makes me seem like I don’t know what I’m doing. I go in there and it’s like, “Oh who are you again?” And the patient will be right next to me while I gotta explain who I am again. And then I remember that I saw this person yesterday, and the day before yesterday, she knows who I am.” – CHW 1, Site 1

Individuals

© 2024 Schriger SH et al. *JAMA Health Forum.*

Leadership at organization (barrier and facilitator)

“Today it feels like they [leadership at organization] still don’t know much about it... Even though there was this big meeting and those periodic emails… I feel like the understanding of the model today isn’t that strong.” -Leader 2, Site 5

“I think it was very helpful to have someone who knew the importance of having a CHW and that definitely took the risk of saying we should bring CHWs into our healthcare system.” – Manager, Site 3

“I think the president’s support of the program has been key. He has believed in this program from ground up, and there have been a lot of furloughs, and he just keeps saying we need your program more than ever now. So I think just buy-in from the board and upper management in the hospital system has helped.” – Manager, Site 2

Characteristics of CHWs (facilitator)

“Because she was Latina, she was able to understand my diet that I eat. Whereas to an American, they really don’t understand that we gotta have our tortillas, we gotta have our beans, we gotta have our rice. And she was able to have me work around my normal diet to where I could eat something else instead.” -Patient 3, Site 5

“They help you by taking the time that the doctor sometimes can’t or won’t. She takes the time to listen to you.” -Patient 3, Site 5

“They’re really able to connect with them at a level where no one else in the system is able to.” -Manager, Site 1

“She’s even offered to find recipes that she said she would even try at home to see if they’re tasty to help with the weight loss.” – Patient 1, Site 2

“Very few people keep to their word. And, I understand that she has a life too, she has a family to take care of – her kids and her husband. And she works and she has the same problems that anyone else does. And yet she’s still out there and it’s like “wow, this lady is like a” – I can’t put it any other way. In my life, she’s been a blessing.” -Patient 1, Site 5

“The way that I like to think about it is sometimes providers or people who are within the hospital walls don’t have the time to go out and see what exactly it is to live this person’s life. So I feel like the CHWs get a little glimpse of what is really going on with the patient, what their real struggles are, not only clinically but socially, emotionally. So it’s definitely a different perspective. And so I think that that makes them so valuable, because they bring like the other side of the story, you know the side that the patient sometimes doesn’t get to tell.” -Manager, Site 3

“I think he’s great. I think he’s got a lot of good ideas. I think that he’s very knowledgeable, very personable. I think he really cares about me and his other patients that he may have.” -Patient 2, Site 4

IMPaCT implementation team (facilitator)

“The folks on the IMPaCT team, you get the sense that they are operators… they’re not the sort of staid academic type that is perhaps a bit too rigid about what real-world conditions might look like.” -Leader 3, Site 1

“I really liked that support, because we had meetings before every two weeks, which was great because we just implemented the program, we were new to this, so just having that guidance for any questions we had if we didn’t understand certain things.” -Manager, Site 5

Implementation process

Program boundaries (barrier)

© 2024 Schriger SH et al. *JAMA Health Forum.*

“They don’t want to talk to anybody else, they just want to talk to you. And so, you become their therapist, you know? And even though we kind of push, “You know, we have this program you can talk to this person” and they’re just like, “No, it’s fine, I’ll just talk to you.” -CHW 3, Site 5

“She would always open up the doors with her cell phone. Always ensured I had her email address like, ‘Don’t worry about the time.’ Always assuring, genuinely, that if I needed to call she was a call away.” -Patient 1, Site 4

“Because of the length of the program, six months, they get really attached to you and they trust you and trust is a big thing. And so sometimes we end up having to be therapists, counselors, and filling out applications, and things like that. You know, things that are just out of our place. Because they won’t take the help from anybody else.” -CHW 3, Site 5

The conversations were scheduled to be half an hour long, sometimes they would stretch 45 minutes, an hour. I think there was one time where our conversation actually lasted – you know when you hang up, you can look at your phone and see how long the call was. One of those phone calls lasted more than 3 hours. – Patient 1, Site 5

(About contact with CHW after official end of program): “I still talk to him sometimes. I call him, I talk to him. I wish he could continue with me.” -Patient 1, Site 1

Training (barrier and facilitator)

We don’t have that training, so we don’t know- other than listening and giving advice- we don’t know what else to do... They said “if you could get another training or one more resource” what would it be? And we all said, “mental health – CHW 1, Site 5

“It was amazing… it made me feel so comfortable taking on this role as a CHW. It made me feel so confident, especially all the role plays we did where we can see all the potential situations with the patient... I’ve never seen any better training for any other jobs. It made me want to be a CHW trainer honestly.” -CHW 2, Site 1

“If we could clone [IMPaCT trainer] or bring her back more often, we would. I don’t think our program would be the same if she hadn’t come in at the beginning and really set the groundwork and the values and the attitude that she did.” – Leader 1, Site 1

“ It was a comprehensive training. I really liked the fact that I was able to shadow a community health worker in the field; that was really impactful for me because I was able to see the work, hands on, that the CHWs were doing.” -Manager, Site 1

“I can be the person who listens but I don’t have the tools that a mental health therapist will have to support them.” -CHW 3, Site 4

Fidelity (barrier and facilitator)

“Our process looks a lot different than the IMPaCT model’s recommendations, just because of our HR restrictions.” -Manager, Site 2

(About IMPaCT recommended hiring practices): “I really used most of the materials that IMPaCT had developed to plan a meet and greet with people in the community… I found it really useful and different than any of the recruitment processes I had either led or been a part of myself, definitely a unique approach to identifying the qualities and traits of individuals, as opposed to, necessarily, their skill sets or past work… looking back, who we ended up hiring and how they differ a little bit from other (Agency Name) staff members that are in similar roles across the system, I really attribute that to the different approach we took to recruit them.” -Leader 2, Site 1

© 2024 Schriger SH et al. *JAMA Health Forum.*

“I thought [the IMPaCT hiring recommendations] were absolutely awesome. I think what made it difficult was the model was, at that point, very different for [our organization] and how our human resource department works” - Leader 2, Site 3

I wish we would’ve had the system that IMPaCT uses since it’s what’s easier and I was trained when we first got trained that way –Manager, Site 5

(About IMPaCT recommended hiring practices): “I have gotten really, really good feedback from the leads involved… they loved the way that it was structured. They said it was—they had never been part of something like that. Yeah, and they really felt like they got to know people on a different level than coming in with a resume, interview questions… it was more about the person itself, and how they interacted, who they were at their core, versus just a resume." –Leader 1, Site 3

© 2024 Schriger SH et al. *JAMA Health Forum.*

eFigure. Frequently Used Descriptors of Community Health Workers

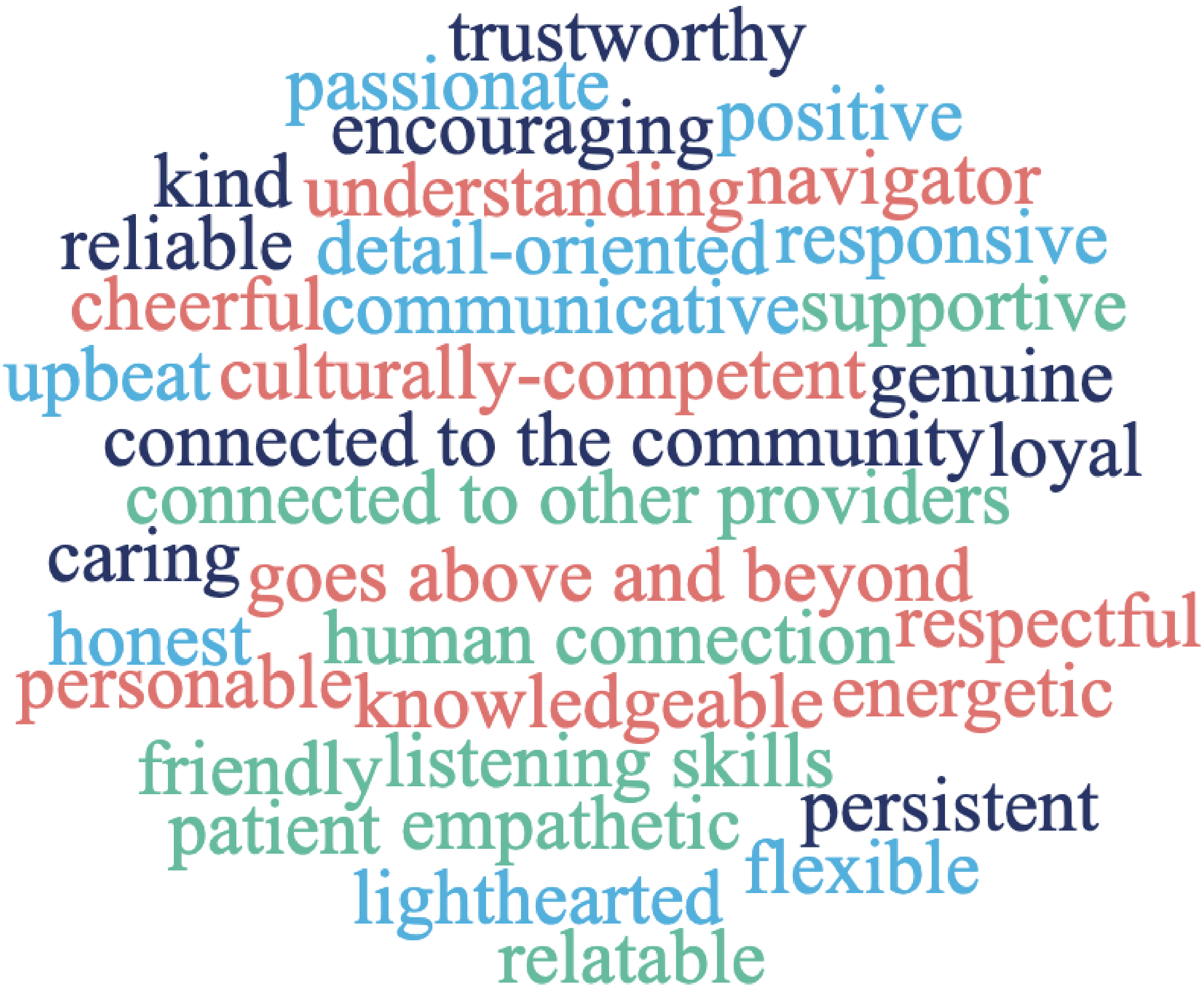

Descriptors commonly used to describe community health workers across interviews with health system leaders, program managers, and patients

© 2024 Schriger SH et al. *JAMA Health Forum.*
