# Supplementary material for: Barriers and Facilitators to Implementing an Evidence-Based Community Health Worker Model
Source: JAMA Health Forum. 2024 Mar 8;5(3):e240034. doi: 10.1001/jamahealthforum.2024.0034 (PMC10924240; doi:10.1001/jamahealthforum.2024.0034)
Supplement: Supplement 2. — Data Sharing Statement [file jamahealthforum-e240034-s002.pdf]

# Data Sharing Statement

Schriger. Barriers and Facilitators to Implementing an Evidence-Based Community Health Worker Model. *JAMA Health Forum*. Published March 08, 2024. doi:10.1001/jamahealthforum.2024.0034

**Data**
**Data available:** No
